# Electron microscopic analysis of necrotic bone and failed implant surface in a patient with medication-related osteonecrosis of the jaw

**DOI:** 10.1186/s40902-023-00402-9

**Published:** 2023-10-03

**Authors:** Ji Young Ha, Mi Young Eo, Buyanbileg Sodnom-Ish, Kezia Rachellea Mustakim, Hoon Myoung, Soung Min Kim

**Affiliations:** 1 Department of Oral and Maxillofacial Surgery, Dental Research Institute, School of Dentistry, Seoul National University, 101 Daehak-Ro, Jongno-Gu, Seoul, 03080 Korea; 2 Oral and Maxillofacial Microvascular Reconstruction Lab, Brong Ahafo Regional Hospital, Ghana Health Service, P.O. Box 27, Sunyani, Brong Ahafo Ghana

**Keywords:** Bisphosphonate-related osteonecrosis of the jaw, Necrotic bone, Failed implant, Medication-related osteonecrosis of the jaw, Energy dispersive X-ray spectroscopy

## Abstract

**Background:**

Bisphosphonates (BP), a commonly used medication for various bone diseases, have been known to have severe complications such as bisphosphonate-related osteonecrosis of the jaw (BRONJ). Failure of dental implants has also been found in patients with medication-related osteonecrosis of the jaw (MRONJ). In this study, we analyzed the necrotic bone tissues and the surface of the failed implants removed from the jaw in patients treated with BPs and antiresorptive agents.

**Results:**

Chronic inflammatory cells with collagen and fibrous tissues and bone sequestrum were shown at 5.0 × , 10.0 × , 20.0 × , and 40.0 × magnified histologic sections in the bone and fibrotic scar tissues removed from patients with MRONJ due to osteonecrosis. Hardened bone tissues with microcracked bony resorbed lacunae were observed in SEM. Unlike the previously published comparative data where immune cells, such as dendritic cells, were found in the failed implant surface, these immune cells were not identified in the BRONJ-related peri-implantitis tissues through the TEM investigations. Furthermore, EDS revealed that in addition to the main titanium element, gold, carbon, oxygen, calcium, phosphorus, silicon, and sulfur elements were found.

**Conclusion:**

Hardened bone tissues with microcracked bony resorbed lacunae were observed in the SEM findings, which were considered as the main characteristic of the osteonecrosis of the jaw. Immune cells, such as dendritic cells were not identified in the TEM. EDS showed that in addition to the main titanium element, gold, carbon, oxygen, calcium, phosphorus, and silicon elements were found. Furthermore, it was revealed that sulfur was found, which was considered to be one of the complicated causes of implant failure in patients with BRONJ.

## Background

Several studies have been conducted to examine the response of the jaw bone in the absence of systemic disease during implant placement. In a patient with severe bruising, the electron microscopic findings of the fractured implant, removed with trephine bur approximately 4 years after implant placement, showed similar results to those of normal bone healing [1]. Bone bonding to nano-textured titanium implant surfaces is promoted in human jawbone after functional loading [2]. In another electron microscopic analysis of the surface of the failed implant removed in patients without systemic disease, the implant surfaces showed new components like calcium (Ca), sodium (Na), and chloride (Cl) elements in trace quantities [3].

In case of patients with compromised systemic conditions, several jaw bone diseases associated with osteonecrosis, including bisphosphonate-related osteonecrosis of the jaw (BRONJ), drug-related osteonecrosis of the jaw, medication-related osteonecrosis of the jaw (MRONJ), jaw osteomyelitis, and osteoradionecrosis of the jaw, have been studied extensively [4–7]. These entities may have a significant impact on the osseointegration of implant fixtures.

BRONJ is one of the complications from the use of bisphosphonates (BP), which is a common medication used for various bone diseases including osteoporosis and bone malignancy [6]. The BP shares a P–C-P structure compared with the P-O-P structure of pyrophosphate. Different BPs vary in the two R groups. Despite their structural similarity, there are important differences in potency and toxicity. Nitrogen-containing BP (N-BP) (zoledronic acid, risedronate, ibandronate, alendronate, neridronate, pamidronate) is a more potent inhibitor of bone resorption than simple BP (etidronate, clodronate, tiludronate) [8, 9].

Many clinicians and researchers have published the cause and clinical or histological findings of BRONJ, until recently. Failure of dental implants has also been found in patients with BRONJ. Various methods have been utilized for analyzing any unusual findings found in the necrotic bone tissues removed from patients with BRONJ and the dental implant surface in comparison to the failed implant in patients without systemic disease [10, 11]. However, in the cases of BRONJ, the ultrastructure of the affected bone and dental implant surfaces has not been studied.

Therefore, in this study, we analyzed the necrotic bone tissues and the surface of the failed implants removed from the jaw of patients treated with BPs for various reasons by using an electron microscope, under the null hypothesis that ultrastructural findings of necrotic bones and failed implant surface in patients with BRONJ are not different from those in patients with peri-implantitis, osteomyelitis, and osteoradionecrosis of the jaw.

## Methods

### Patients data

We reviewed a total of 5 patients diagnosed with BRONJ who visited Seoul National University Dental Hospital (SNUDH, Seoul, Republic of Korea) and treated clinically. The current study and its access to patient records were ethically approved by the Seoul National University Institutional Review Board (S-D20200007). The study was conducted in accordance with the relevant guidelines and regulations of the Declaration of Helsinki, and written informed consent was obtained from all participants. The MRONJ diagnosis was established through patient medical history, clinical evaluation, radiologic findings, and histopathologic examination. The stage for MRONJ was determined according to the American Association of Oral and Maxillofacial Surgeons (AAOMS) guidelines [6, 7].

Inclusion criteria of these patients were (1) patients with MRONJ who were also diagnosed and previously or currently treated for osteoporosis with BPs and other antiresorptive drugs; (2) patients with full clinical data, including periodic follow-up (immediately, 1 month, 3 months, 6 months, and 1 year after surgery) panoramic radiographs and laboratory data results. Exclusion criteria were (1) patients with BRONJ who were also diagnosed and previously or currently treated for multiple myeloma, breast cancer, and other malignancies with BP and (2) patients with incomplete medical records, such as the name of BP, BPs, and dosage and duration of treatment of other antiresorptive drugs, laboratory data, or periodic radiographs, and those who were lost during follow-up.

### Specimen collection

Following the removal of the implant and surrounding tissue, the specimens were grasped with sterile surgical forceps or pincette without any contamination to other material and were immediately put inside the conical tube containing 2.5% glutaraldehyde (GA).

### Specimen processes for histological and transmission electron microscope (TEM) analysis

Every specimen from each patient was decalcified with a solution of 0.5 M ethylene diamine tetra-acetic acid (pH 8.0) (0.5 M EDTA, pH 8.0; BIOSESANG, Sungnam, Korea), dehydrated with 70% ethanol, and embedded into paraffin blocks. The 4-μm-thick slides were then washed with xylene for approximately 10 min and were stained with hematoxylin and eosin. The slides were analyzed with a light microscope (OLYMPUS BX41®; OLYMPUS, Tokyo, Japan). For TEM analysis, histologic sections were fixed in 2.5% glutaraldehyde, stripped into 1 × 1 × 1 mm blocks, embedded in epoxy resin, and cut into ultrathin 70–80 nm sections by continuing scanned under 3000 × , 6000 × , and 10,000 × magnifications by TEM examination (JEM-1400 Flash®, Jeol Ltd., Tokyo, Japan).

### Specimen processes for SEM–EDS analysis

The dental implants with its surrounding tissues were immediately placed in a solution of 2.5% GA in 0.1 M of phosphate buffer after irrigation. The implant fixtures underwent coating before scanning electron microscope (SEM) examination (JSM-7800F Prime®; Jeol Ltd., Tokyo, Japan). The dental implants and its surrounding tissue were analyzed in the upper, middle, and apical parts of the fixture. The surface was scanned thoroughly under 500 × magnification, and areas with representative features were chosen for ultrastructure and element analysis. The SEM was operated at 10 kV, and 65 × , 500 × , 1000 × , 2500 × , 5000 × , 10,000 × , and 20,000 × micrographs were acquired.

Qualitative and semi-quantitative elemental analysis, including element distribution mapping, was performed with an energy dispersive X-ray spectroscopy (EDS) instrument (XFlash® 6, Bruker, Berlin, Germany) connected to a microscope detector and the ESPRIT® analysis software (Bruker, Berlin, Germany). Relative concentrations were indicated by color density in the distribution maps. Representative points in each region were chosen and analyzed under 10,000 × magnification.

## Results

### Analyses of patients data (Table 1)

**Table 1:** Summarized patients’ data

Patients	Age/sex	Jaw	Location	Symptoms	1st drug		2nd drug		Disease stage	Surgical procedures
A	79/F	Mx	Ant	Pain on extractions	Risedronate	7 years	Ibandronate	3 years	2	Saucerization following bone graft
B	59/F	Mn	Post	Persistent pain on implants	Bazedoxifene	1 years	Denosumab	Recently	3	Saucerization following bone graft
C	64/F	Mx	Post	Peri-implantitis/sinusitis	Alendronate/Risedronate	2 years	Zolendronic acid	4 years	2	Saucerization following bone graft, implants
D	79/F	Mx	Post	Rheumatism/sinusitis	Arcoxia/Risedronate/cholecalciferol	4 years	-		3	Modified endoscopic sinus surgery following bone graft
E	68/F	Mx	Post	Peri-implantitis	Risedronate	3 years	-		2	Saucerization following bone graft, implants

*Mx* maxilla, *Mn* mandible, *Anterior* Ant, *Posterior* posterior

#### A 79-year-old female patient with a lesion in the maxillary anterior area (Patient A)

A 79-year-old female patient was referred due to delayed healing and severe bone loss in the extraction socket area of teeth #12 and 13. The patient had symptoms of periodontitis in the #12–13 areas, and 11 months after the extraction of these teeth, swelling persisted with delayed healing. The patient had a medical history of osteoporosis and had been taking 35 mg of risedronate sodium once per week and vitamin D every week for 7 years with 3 mg of ibandronic acid injection every 3 months for the past 3 years.

**Fig. 1:**
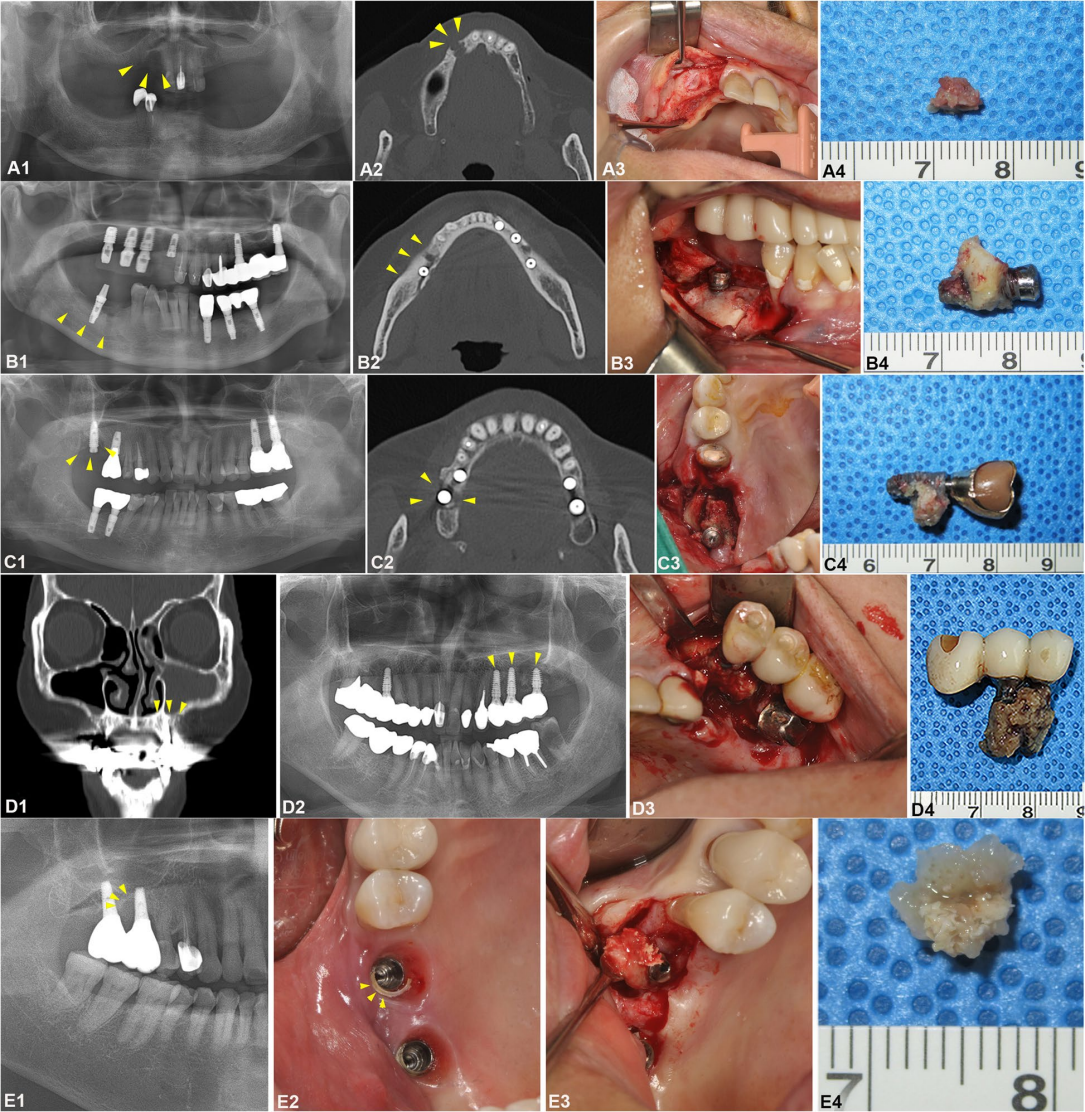
Preoperative radiographic findings of patient A showed the osteolytic lesion in the #13 tooth area (arrows) (A1, A2). Preoperative radiographic findings of patient B showed sequestrum formation in the #46i implant area, with sclerotic change in the #45 and 46 areas with thickening of mandibular bone (arrows) (B1, B2). Preoperative radiographic findings of patient C showed a peri-implant bone loss in the #17i implant area, and maxillary sinus atrophy, but with no significant pathological findings and was ruled out as localized osteomyelitis (MRONJ) (arrows) (C1, C2). Preoperative radiographic findings of patient D showed mucosal thickening on both maxillary sinuses, with alveolar bone fracture, sequestrum, increased marrow attrition, and bone sclerosis on both sides of the maxilla on #15i, #16i, and #24i-26i (arrows) (D1, D2). The panoramic radiograph of patient E showed marginal bone loss (arrows) in the #16i and #17i areas (E1). The excess cement was observed following the crown removal (arrows) (E2). The intraoperative views of the saucerization procedure to remove the main mass (A3, B3, C3, D3, E3). Obtained specimens for further ultrastructural analysis (A4, B4, C4, D4, E4)

The patient had a chief complaint of bleeding and swelling in the extraction socket area. Upon clinical examination, gingival swelling and redness were observed in the #12–13 areas. The panoramic and Waters’ views showed loss of teeth #12–17, 23–27, 37–47, and round-shaped radiolucency observed in #12 area where the root of the tooth was located (Fig. 1A1). The computed tomography (CT) showed an osteolytic lesion with a weak lobulated margin observed in the area where the root of tooth #13 was located (Fig. 1A2).

Based on the abovementioned findings, a diagnosis of BRONJ was made. To perform surgical procedures including sequestrectomy under cooperation with the endocrinology department, BP was suspended for 100 days, and alternative drugs were prescribed for osteoporosis treatment at the same time. Four weeks before the surgical procedure, 250 mg of Celecoxib, a non-steroidal anti-inflammatory drug; 250 mg of Cephalosporin, a second-generation cephalosporin antibiotic; and 15 mg of Lansolazole were prescribed once a day with 400 IU of tocopherol and vitamin B complex once a day for 3 months. After a suspension of BP for 100 days, the patient underwent saucerization, biopsy, and bone grafting using allogenic bone (Oragraft®, Lifenet, VA, USA) and xenogeneic bone (Bio-Oss®, Geistlich Biomaterials, Wolhusen, Switzerland) in the #12 and 13 areas under conscious sedation using 5 mg midazolam (Fig. 1A3–A4). The patient underwent uneventful healing postoperatively and was closely followed.

#### A 59-year-old female patient with a lesion in the mandibular posterior region (Patient B)

A 59-year-old female patient was referred to our hospital due to persistent pain in the right mandible, tongue, cheek, and ear. The patient received implant installation in #45 area 5 months ago and experienced pain and slight paresthesia in the right mandible. The symptoms improved after the removal of the implant 5 days after installation. However, 3 months after implant removal, the patient suffered from pain in the right mandible area again. The patient had a medical history of osteoporosis and was taking 20 mg of Bazedoxifene for 1 year followed by 60 mg/ml of Denosumab injection once before visiting our hospital.

Clinically, swelling and redness were observed in the right mandible area and pus discharge from the #46 implant sulcus. Radiographic examination showed sequestrum formation in the #46 implant area, with sclerotic change in the #45–46 areas and thickening of mandibular bone (Fig. 1B1–B2).

Based on the history, clinical, and radiographic findings, a diagnosis of MRONJ was established. The patient was treated with saucerization in the right posterior mandible area with #46 implant removal under conscious sedation using 5 mg midazolam (Fig. 1B3–B4). The patient was prescribed with 250 mg of Celecoxib, 250 mg of Cephalosporin, and 15 mg of Lansolazole 3 times a day with 400 IU of tocopherol and vitamin B complex once a day for 3 months. The biopsy results showed chronic suppurative osteomyelitis and clinically MRONJ. The patient showed uneventful healing during the follow-up period. Six months later, the patient underwent a bone grafting procedure in the right posterior mandible area with allogenic bone (Oragraft®, Lifenet, VA, USA) and xenogeneic bone (Bio-Oss®, Geistlich Biomaterials, Wolhusen, Switzerland) by subvestibular approach. Four months after the bone grafting procedure, the patient received implant installation in #45, 46, and 47 and was followed up closely.

#### A 64-year-old female patient with a lesion in the maxillary posterior region (Patient C)

A 64-year-old female patient was referred for evaluation and management of #17 implant peri-implantitis and right maxillary sinusitis under the suspicion of BRONJ. The patient had a history of osteoporosis and was taking 70 g of Alendronate and 35 mg of Risedronate sodium once a week for 2 years followed by 5 mg of Zolendronic acid injection once every year for 2 years and then every 6 months for 2 years.

The clinical examination showed swelling and redness in the #17 implant area. The radiograph showed a peri-implant bone loss in the #17 implant area and maxillary sinus atrophy with no significant pathological findings so the clinical diagnosis of localized osteomyelitis of BRONJ was made (Fig. 1C1–C2).

Based on the clinical, and radiographic findings, the patient was diagnosed with BRONJ. Preoperatively, 250 mg of cephalosporin, 386 mg ibuprofen arginine, and 95 mg Phazyme were prescribed three times a day for 5 days and 400 IU of tocopherol and vitamin B complex once a day for 3 months before surgery. The patient was treated with saucerization in the right upper maxilla, removal of #16 and 17 implants under conscious sedation using 5 mg of midazolam (Fig. 1 C3–C4). The biopsy results confirmed sequestrum in the #16 implant area. The patient had uneventful healing and was closely followed up every week, then by every month, every 3 months, and every 6 months.

#### A 79-year-old female patient with a lesion in the maxillary posterior region (Patient D)

A 79-year-old female patient was referred from another hospital under the suspicion of BRONJ. The patient had been suffering from rheumatism for 20 years, in addition to osteoporosis and heart valve defects. The patient had been taking Arcoxia and risedronate/cholecalciferol 35 mg/5600 IU tab once per week for the past 4 years. From the clinical examination, there was buccal bone exposure on the #16 and #26 implant areas. The radiograph showed mucosal thickening on both maxillary sinuses, with alveolar bone fracture, sequestrum, increased marrow attrition, and bone sclerosis on both sides of the maxilla and left mandible (Fig. 1D1–D2). Based on the clinical and radiographic examination, BRONJ was confirmed.

The patient was prescribed with Feroba 256 mg once a day for 12 weeks. The patient discontinued BP medication for 2 months which was substituted with a daily teriparatide injection. Under intravenous sedation, the modified endoscopic sinus surgery with partial maxillectomy on the left maxillary was performed. The removed main mass was sent for biopsy, and the results were chronic peri-implantitis, fungal sinusitis, and sequestrum. The patient was followed regularly and managed with sinus irrigation. After 3 months, saucerization, mass excision, and nerve decompression were performed on the left mandible with biopsy results confirming chronic osteomyelitis with sequestrum. Seven months later, mass excision was performed on the right maxilla together with implant removal, and the biopsy result was confirmed to be sequestrum (Fig. 1D3–D4). The patient was followed up and managed with simple curettage every 2 to 3 months with additional saucerization and bone grinding on the left mandible. A biopsy was performed after the left mandible curettage to confirm the extension of the lesion until no necrotic bone was observed, and a bone graft was performed afterward. With the appropriate and routine management, the radiogram showed normalization of the maxillary sinus and uneventful healing of the left mandible.

#### A 68-year-old female patient with a lesion in the maxillary posterior region (Patient E)

A 68-year-old female patient was referred with grade 1 mobility on #16 and grade 3 mobility on #17. The patient had osteoporosis and was taking Risedronate 35 mg once a week during the last 3 years. No specific finding was observed in the laboratory blood test result. Due to the severe mobility of #17, the tooth was extracted along with #16, and implant installation was planned. The patient was instructed to discontinue the BP medication for 6 months before extraction of #16. The extraction site of #16 showed uneventful healing, and the biopsy result was chronic periodontitis. Four months after the extraction, sinus lifting with implant installation on #16 and #17 areas was performed.

The sinus lifting on the right maxilla was performed through the lateral approach. After creating a lateral bony window using a round bur, the Schneiderian membrane was exposed and elevated using a sinus kit. The patient’s bone was crushed, and together with allogeneic particulate bone, Oragraft® (LifeNet Health Co., VA, USA) was used as bone graft material for sinus lifting. Implant installation was done using 4.0 × 8.5 mm Stella® implant fixtures (Shinhung Co., Seoul, South Korea) for #16 and #17. Six months later, two 4-mm regular platform (RP) healing abutments (HA) were placed followed by prosthesis delivery 3 months later.

At a 1.5-year follow-up, the patient presented with gingival erythema and pus discharge from the #16i and #17i areas. The panoramic radiograph showed marginal bone loss, suggestive of peri-implantitis (Fig. 1E1). The #16 and #17 implant crowns were removed. Following the removal of crowns, excess cement was found surrounding the implant fixture (Fig. 1E2). The cement was removed and curettage surrounding implant fixtures with minocycline application was performed, and granulation tissue from #16 implant area was curetted along with the bone under local anesthesia after one month (Fig. 1E3–E4), followed by bone graft using Oragraft® and cover screw replacement. Three months later, re-entry was performed using 4-mm Stella® RP HA on #16 implant and 3-mm RP HA on #17 implant. Prostheses were delivered 3 months later.

### Histological and TEM analysis

**Fig. 2:**
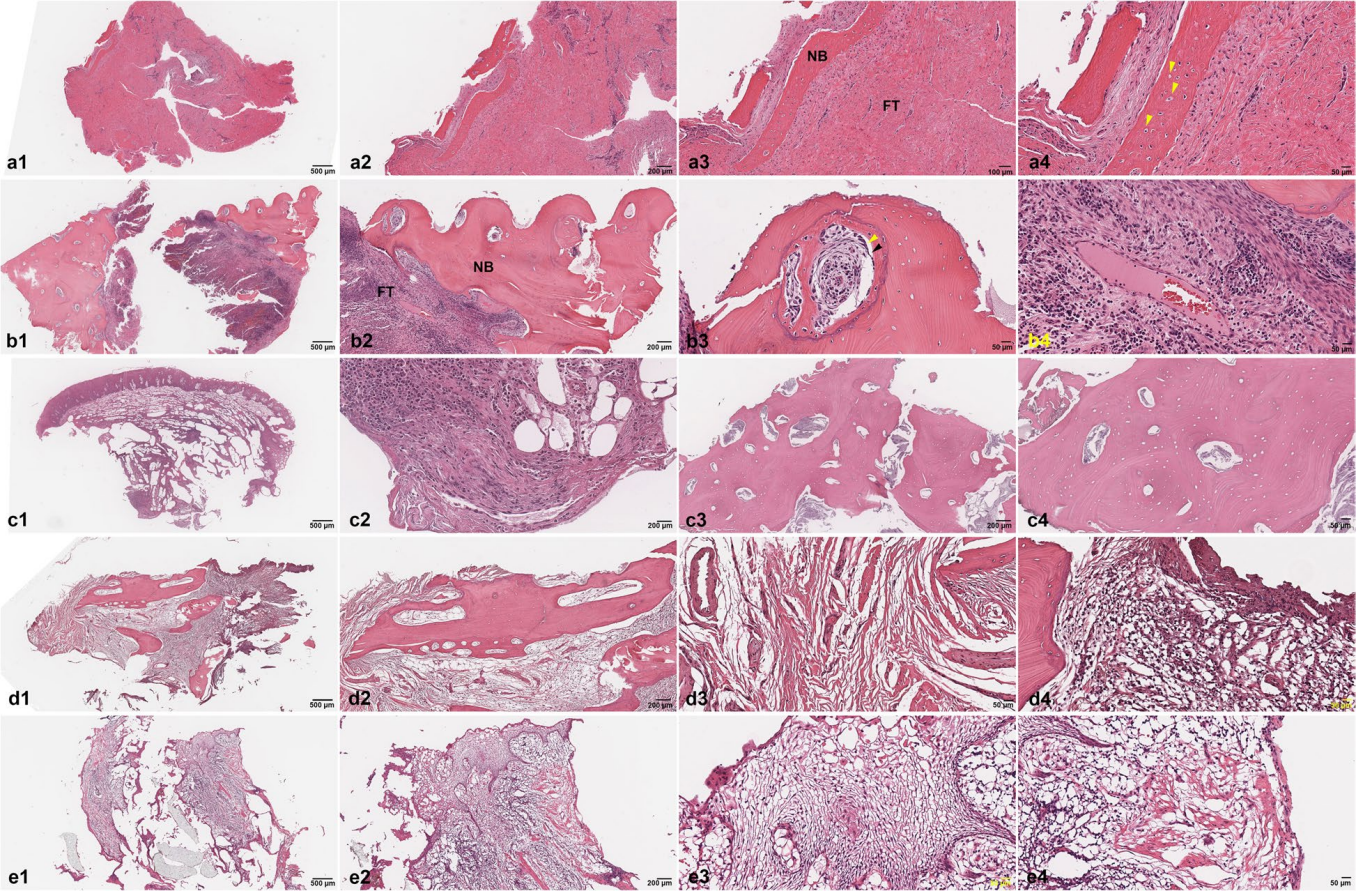
Histopathological findings of specimens in patient A (a1–a4), patient B (b1–b4), patient C (c1–c4), patient D (d1–d4), and patient E (e1–e4). At 10 × magnification, 100 μm, fibrotic tissue (FT), and necrotic bone (NB) can be seen (a3). At magnification 20x, 50 μm, pyknotic osteocytes, and empty lacunae are observed (yellow arrows, a4). At 20 × magnification, 50 μm, osteoblast (black arrow) is detached from the NB (yellow arrow, b3). At 20 × magnification, 50 μm, the fibrous tissue can be seen filled by a plethora inflammatory cells. An oblique view of the venule can also be seen in this section (b4). Fibrotic tissue filled by inflammatory cells can be seen at magnification 2x, 500 μm (c1), and at 5 × magnification, 200 μm (c2). NB with empty lacunae and no sign of osteoblast can be seen at 5 × magnification, 200 μm (c3) and 20 × magnification, 50 μm (c4). Sequestrum/NB, 5 × magnification, 200 μm (d2), fibrotic tissue, 5 × magnification, 200 μm (d3), and inflammatory cells occupy the soft tissue at 20 × magnification, 50 μm (d4). At 20 × magnification, 50 μm, necrotic soft tissue shows the swelling of desmosome in the epithelium (e3) and fibrotic loose connective tissue (e4)

The overall tissue specimen showed a progressive accumulation of collagen, which is considered to be the hallmark of tissue fibrosis shown at low magnification. The basic histopathological characteristics of BRONJ with bone sequestrum were shown at various degrees of magnifications in all cases (Fig. 2). The presence of chronic inflammatory cell infiltration, presented by lymphocyte and plasma cells and osteocyte-depleted bone lacunae along with pyknotic osteocytes, was found at high magnification.

**Fig. 3:**
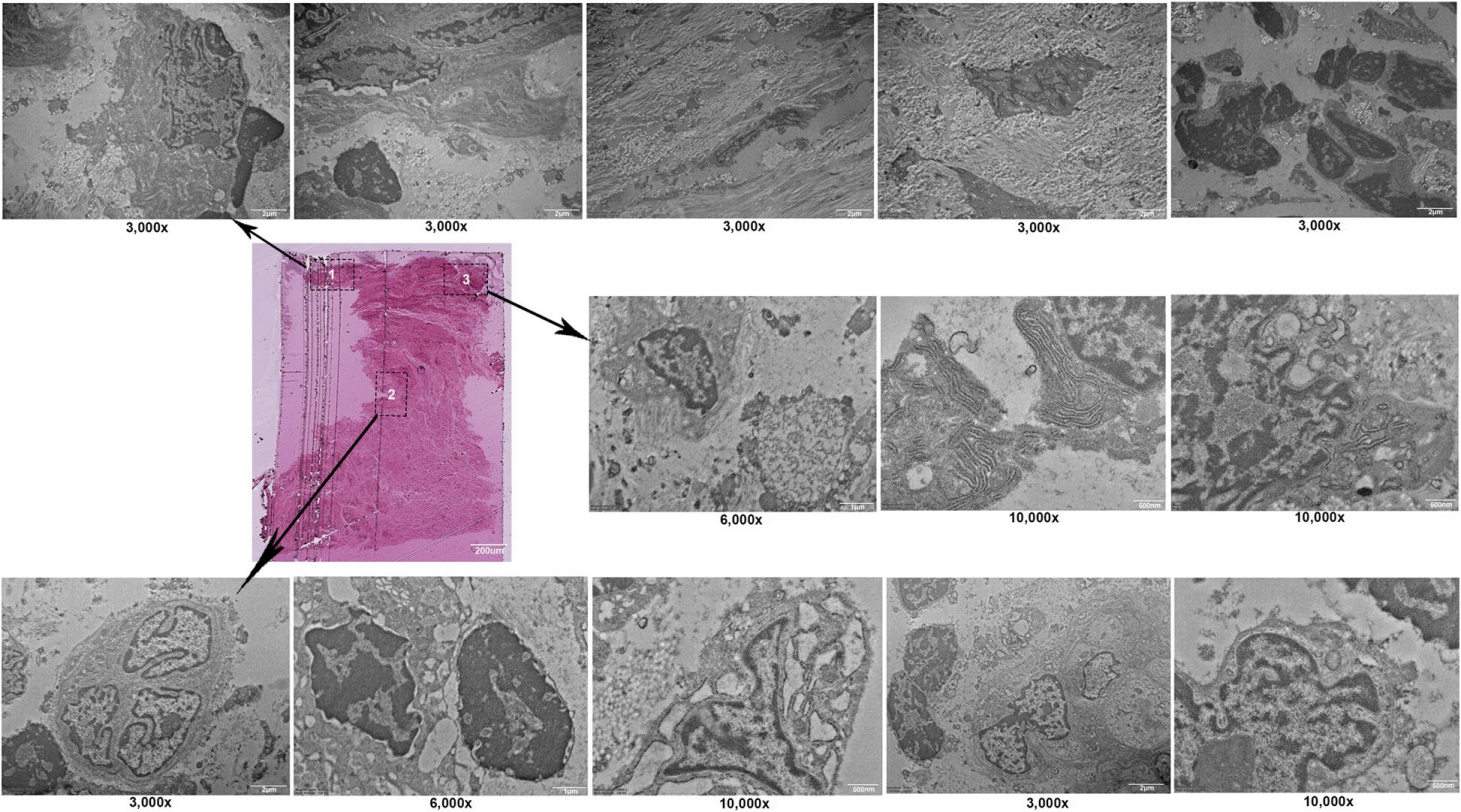
TEM findings of patient A show the cellular composition of the fibrotic scar tissue rich in inflammatory cells, fibrin, and collagen tissue. Macrophage, lymphocyte, and plasma cells were also found. Cells undergoing degeneration and cell death with lipid formation were observed

**Fig. 4:**
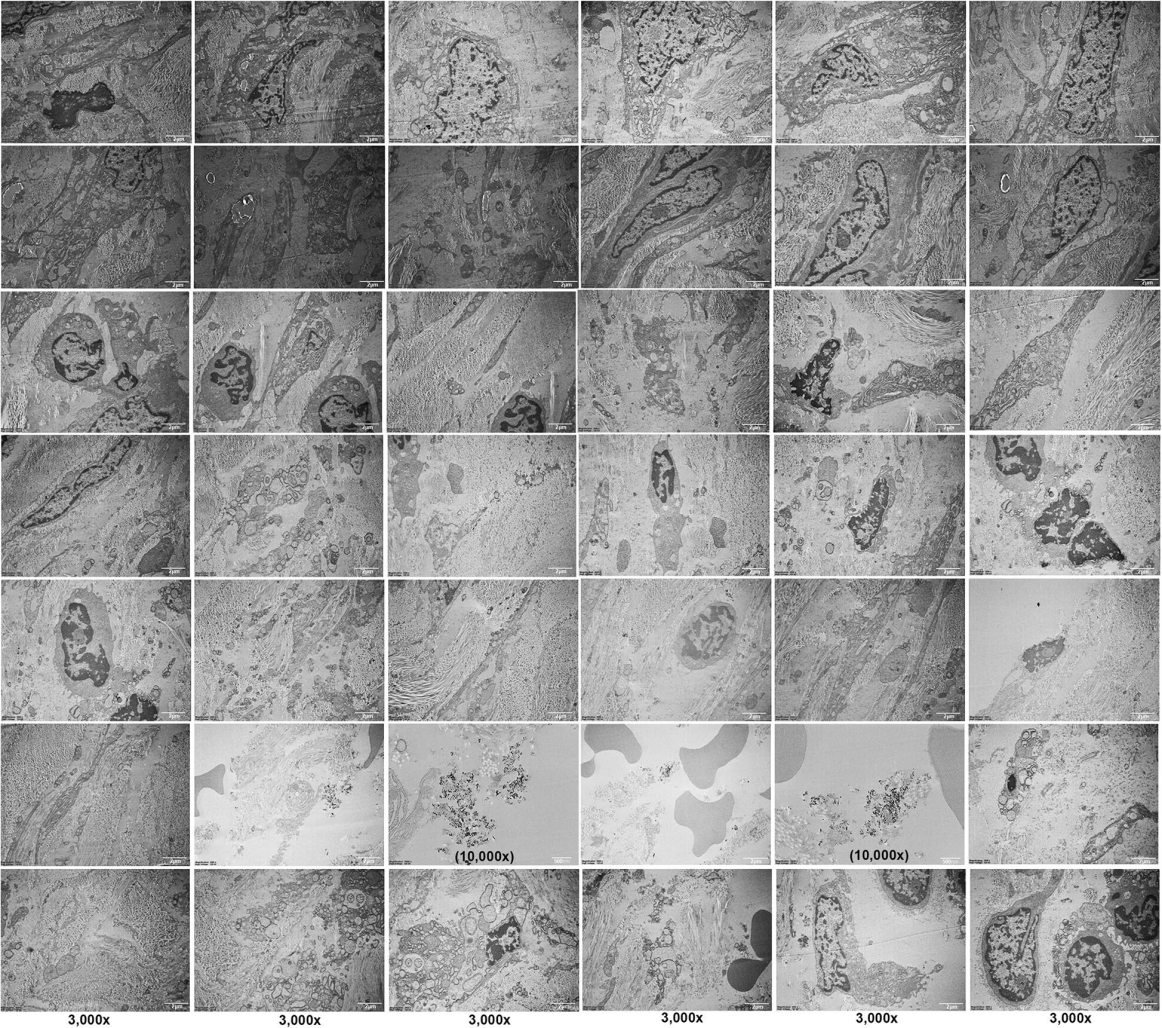
TEM findings of patient E showed various inflammatory cells, including neutrophils, macrophages, and mast cells, and mostly connective tissue rich in fibroblast cells producing type I and type II collagen fibrils

**Fig. 5:**
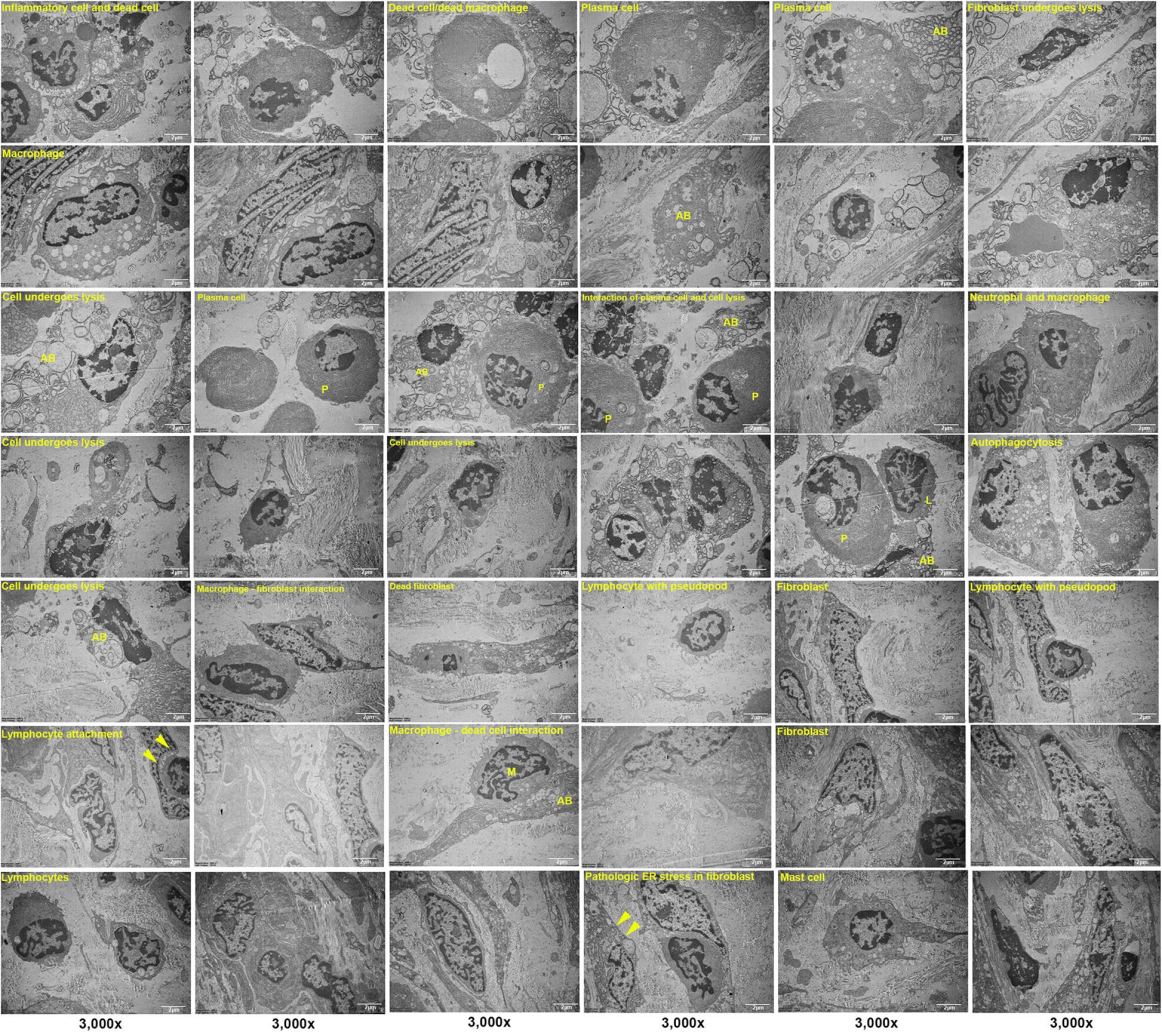
TEM findings of patient E show fibroblast cells undergoing degenerative changes, interaction of plasma cell (P), neutrophil, macrophage (M) with apoptotic bodies of dead cell (AB), mast cell, cell undergoes lysis, autophagocytosis, pathologic endoplasmic reticulum (ER) stress, and lymphocyte attachment with cell can be seen

TEM examination showed the cellular composition of the fibrotic scar tissue rich in inflammatory cells, fibrin, and collagen tissue. The TEM images showed abundant fibroblast cells producing type I and type 2 collagen fibrils. Macrophage, lymphocyte, and plasma cells were also found. Cells undergoing degeneration and cell death with lipid formation were observed in patient A (Fig. 3). TEM findings of peri-implantitis showed fibroblast cells and various inflammatory cells including neutrophils, macrophages, and mast cells (Fig. 4) in patient E. The specimen mostly consisted of connective tissue rich in fibroblast cells producing type I and type II collagen fibrils. Cells undergoing degenerative changes were also found in patient E (Fig. 5), and thus, we defined necrotic bone from these chronic inflammation cells’ infiltrated bony tissues.

### SEM–EDS analysis (Table 2)

**Fig. 6:**
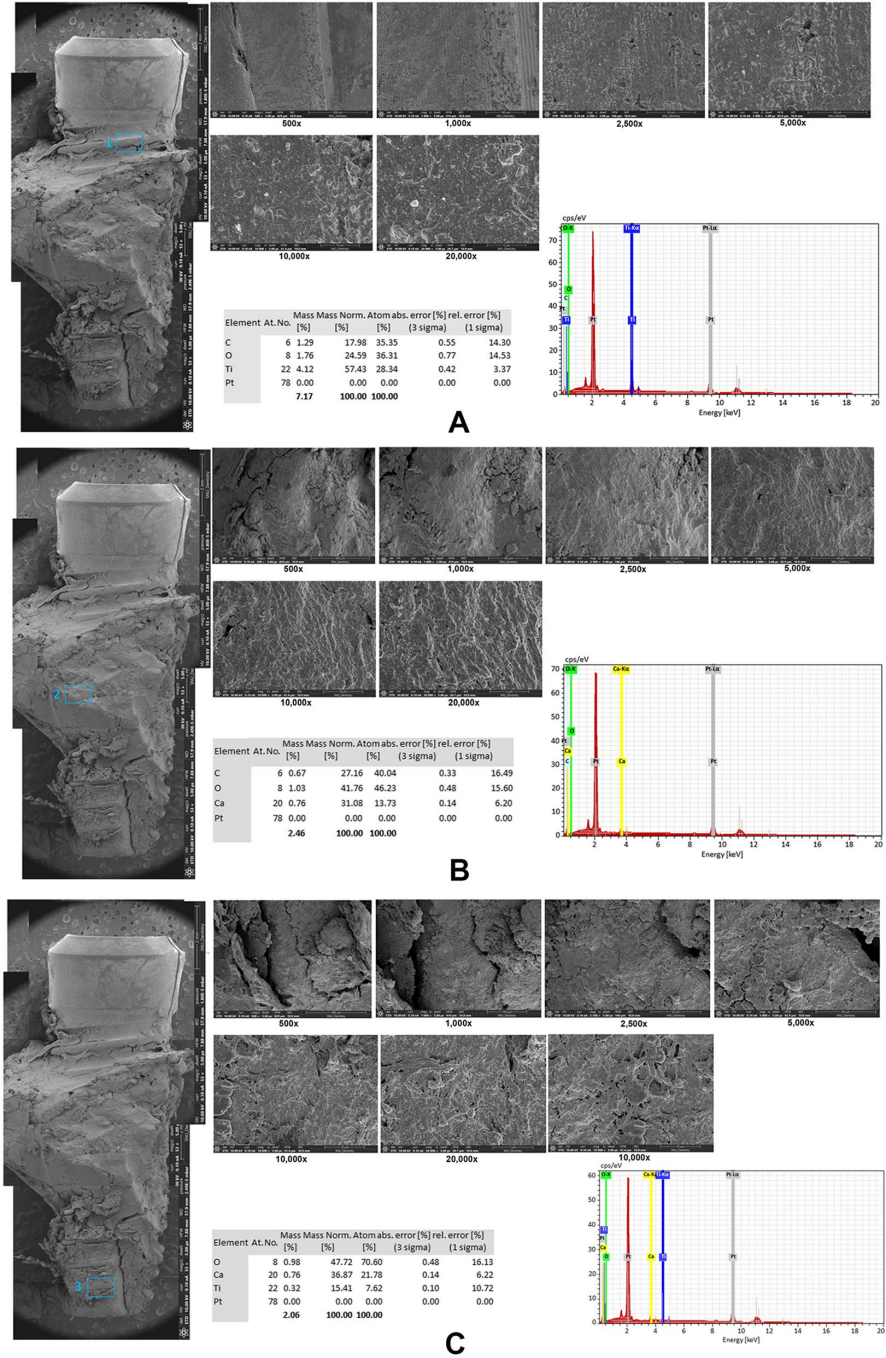
SEM and EDS findings of patient B. The upper region of implant surface in the first thread area showed the typical pattern of sandblasting and acid etching surface. The EDS results showed high levels of titanium (Ti) (57.43%), followed by oxygen (O) (24.59%) and carbon (C) 24.59% (**A**). The attached bone surface of the middle region of the implant, and apical region showed the necrotic bony microcracks lacking any living osteocyte lacunae. EDS results showed high levels of O (41.76%), Ca (31.08%), and C (27.16%) in the middle part (**B**) and high levels of Ti (15.41%), Ca (36.87%), and O (47.72%) in apical part (**C**)

SEM and EDS findings of the implant and the surrounding necrotic bone in patient B were carried out at three points (Fig. 6): (1) the upper region of the implant surface in the first thread area, (2) the attached bone surface in the middle region of the implant, and (3) apical region of the implant surface. The exposed implant surface of point 1 showed the typical pattern of sandblasting and acid etching surface. The EDS results showed a high level of titanium (Ti) (57.43%), followed by oxygen (O) (24.59%) and carbon (C) 24.59% (Fig. 6A). Points 2 and 3 showed necrotic bony microcracks lacking any living osteocyte lacunae. The EDS results of point 2 showed a high level of O (41.76%), Ca (31.08%), and C (27.16%) (Fig. 6B). High levels of Ti (15.41%), Ca (36.87%), and O (47.72%) were detected in point 3 (Fig. 6C).

**Table 2:** EDS results of patients B, C, and D

Region	Elements	B patient		C patient		D patient	
		Weight percentage (wt%)	Atom percentage (at%)	Weight percentage (wt%)	Atom percentage (at%)	Weight percentage (wt%)	Atom percentage (at%)
A (upper region)	C	17.98	35.35	64.34	82.77		
	O	24.59	36.31	11.62	11.23	31.06	54.52
	Ti	57.43	28.34	0.13	0.04	1.01	0.59
	Ca	~	~	8.80	3.39	40.75	28.55
	Si	~	~	2.94	1.62	7.14	7.14
	Au	~	~	12.16	0.95	14.17	2.02
	F	~	~	~	~	~	~
	Na	~	~	~	~	5.87	7.18
B (middle region)	C	27.16	40.04	11.65	27.14	~	~
	O	41.76	46.23	19.25	33.66	12.57	32.41
	Ti	~	~	44.01	25.71	67.03	57.77
	Ca	31.08	13.73	1.84	1.28	1.71	1.76
	Si	~	~	4.41	4.39	1.90	2.79
	Au	~	~	14.97	2.13	15.68	3.29
	F	~	~	3.87	5.70	~	~
	Na	~	~	~	~	1.10	1.98
C (apical part)	C	~	~	22.01	47.61	22.13	59.18
	O	47.72	70.60	20.99	34.07	7.89	15.84
	Ti	15.41	7.62			0.15	0.10
	Ca	36.87	21.78	10.44	6.77	4.11	3.30
	Si	~	~	6.82	6.30	5.66	6.48
	Au	~	~	39.75	5.24	56.57	9.22
	F	~	~	~	~	3.48	5.88
	Na	~	~	~	~	~	~

**Fig. 7:**
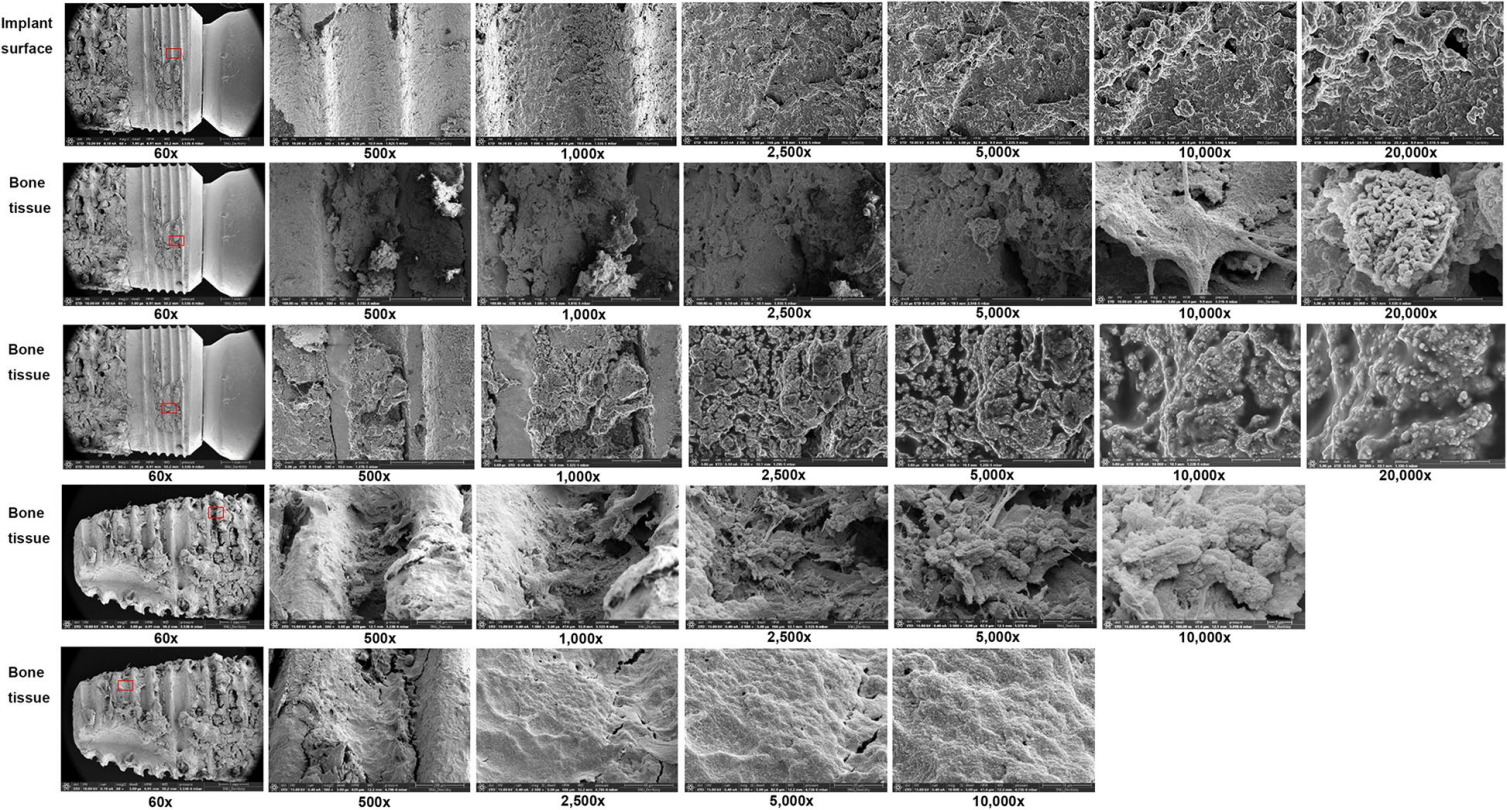
Surface topography of the exposed implant surface to reveal implant surface morphology and its surrounding bony tissue of patient C show typical patterns of sandblasting and acid etching surface (point 1), a bacterial biofilm at 20,000 × magnification (point 2), a more compact sclerosing bony structure (point 3), filamentous structures (point 4), and a microcrack on the necrotic bony tissue (point 5)

**Fig. 8:**
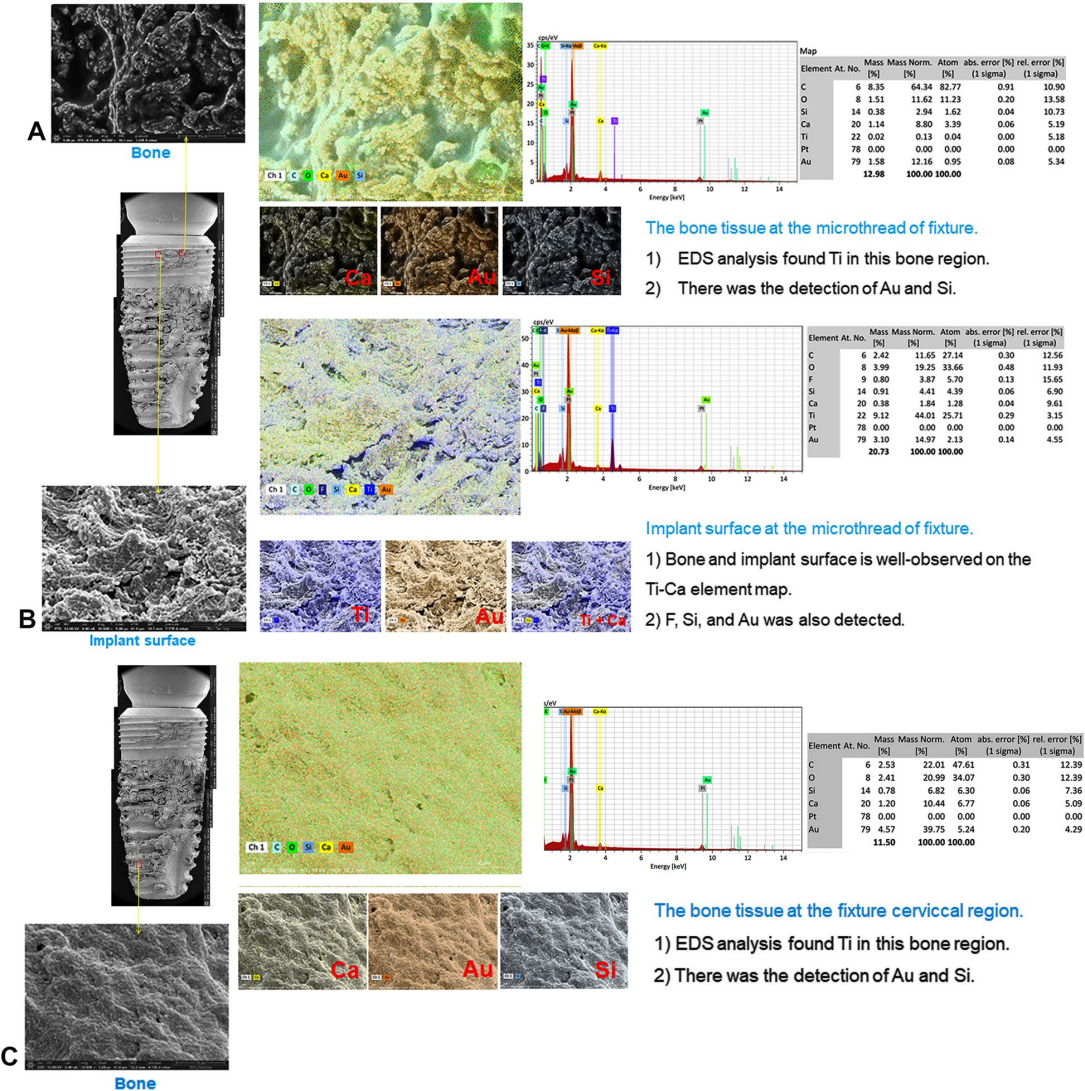
EDS results of the bone tissue at the micro thread of patient C fixture showed high levels of C (% mass: 64.34%) and O (% mass: 11.62). Traces of calcium (Ca) (% mass 8.80%), Ti (% mass Ti: 0.13%), gold (Au) (% mass Au: 12.16%), and silicon (Si) (% mass Si: 2.94%) were found (**A**). The EDS results on the exposed implant surface at the micro thread of the fixture in the upper region of the implant showed a high level of Ti (% mass: 44.01%) (**B**). The bone tissue in the fixture cervical region showed high levels of Au (% mass: 39.75%), C (% mass: 22.01%), O (% mass: 20.99%), and Ca (% mass: 10.44%). Traces of Si (% mass: 6.82%) were also found (**C**)

The investigation of the surface topography and EDS analysis on the dental implant and its surrounding bony tissue of patient C were carried out. The high magnification micrograph was performed at five points: (1) at the exposed implant surface to show implant surface morphology, (2) at the bone tissue in the upper region of the implants showing several microcracks, (3) at the bone tissue located between the threads in the upper region of the implants showing several microcracks, (4) at the bone tissue between the threads in the middle region of the implant, and (5) at the bone tissue located between the threads in the apical region of the implant showing several microcracks and were examined under 60 × , 500 × , 1000 × , 2500 × , 5000 × , 10,000 × , and 20,000 × magnifications. SEM micrograph of the exposed implant surface on point 1 showed typical patterns of sandblasting and acid etching surface (Fig. 7). On point 2, a bacterial biofilm was observed at 20,000 × magnification. On point 3, a more compact sclerosing bony structure was found. On point 4, filamentous structures were seen. On point 5, a microcrack was seen in the necrotic bone tissue. The EDS results of the bone tissue at the micro thread of the fixture showed a high level of C (% mass: 64.34%) and O (% mass: 11.62). Traces of Ca (% mass 8.80%), Ti (% mass Ti: 0.13%), gold (Au) (% mass Au: 12.16%), and silicon (Si) (% mass Si: 2.94%) were found. The EDS results on the exposed implant surface at the micro thread of the fixture in the upper region of the implant showed a high level of Ti (% mass: 44.01%) (Fig. 8). The bone and implant surface were well observed on the Ti-Ca element map. Fluoride (F) (% mass: 3.87%), Si (% mass: 4.41%), and Au (% mass: 14.97%) were also detected. The bone tissue in the cervical region of the fixture showed a high level of Au (% mass: 39.75%), C (% mass: 22.01%), O (% mass: 20.99%), and Ca (% mass: 10.44%). Traces of Si (% mass: 6.82%) were also found (Fig. 8).

**Fig. 9:**
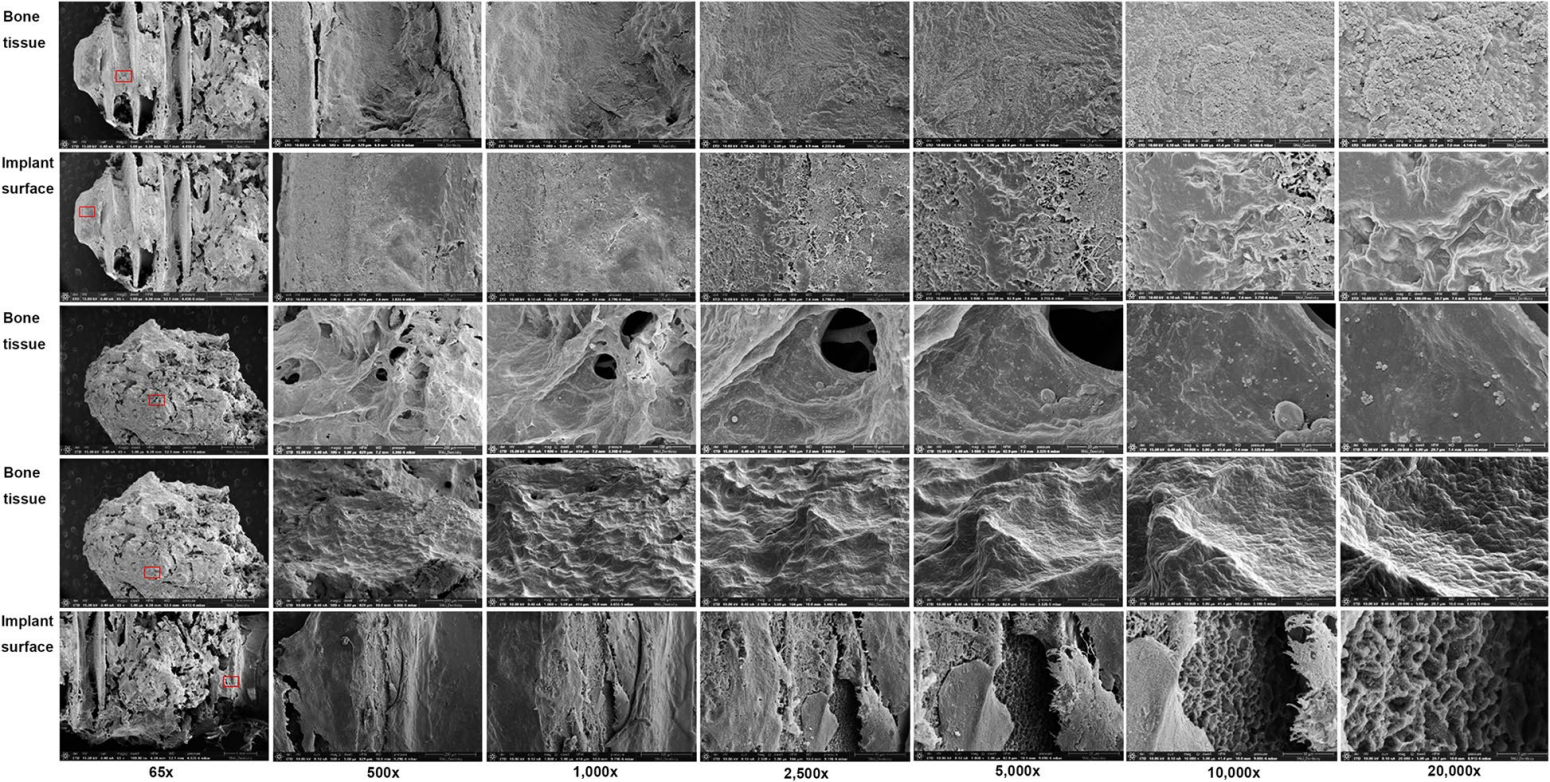
SEM findings of patient D showed five different points: (1) at the bone tissue in the apical region of the implant, (2) at the exposed implant surface in the apical region of the implant, (3) at the necrotic bone tissue attached to the implant, (4) at another necrotic bone tissue attached to the implant, and (5) at implant surface near the upper region of the implant by examination under 65 × , 500 × , 1000 × , 2500 × , 5000 × , 10,000 × , and 20,000 × magnifications. Points 1, 2, and 3 showed typical features of MRONJ with concave areas that show bone resorption pits seen on the bone surface of necrotic bone. The attached necrotic bone tissue on the implant surfaces of points 2 and 3 showed a pattern of sclerosing bone with rare signs of bone lacunae

**Fig. 10:**
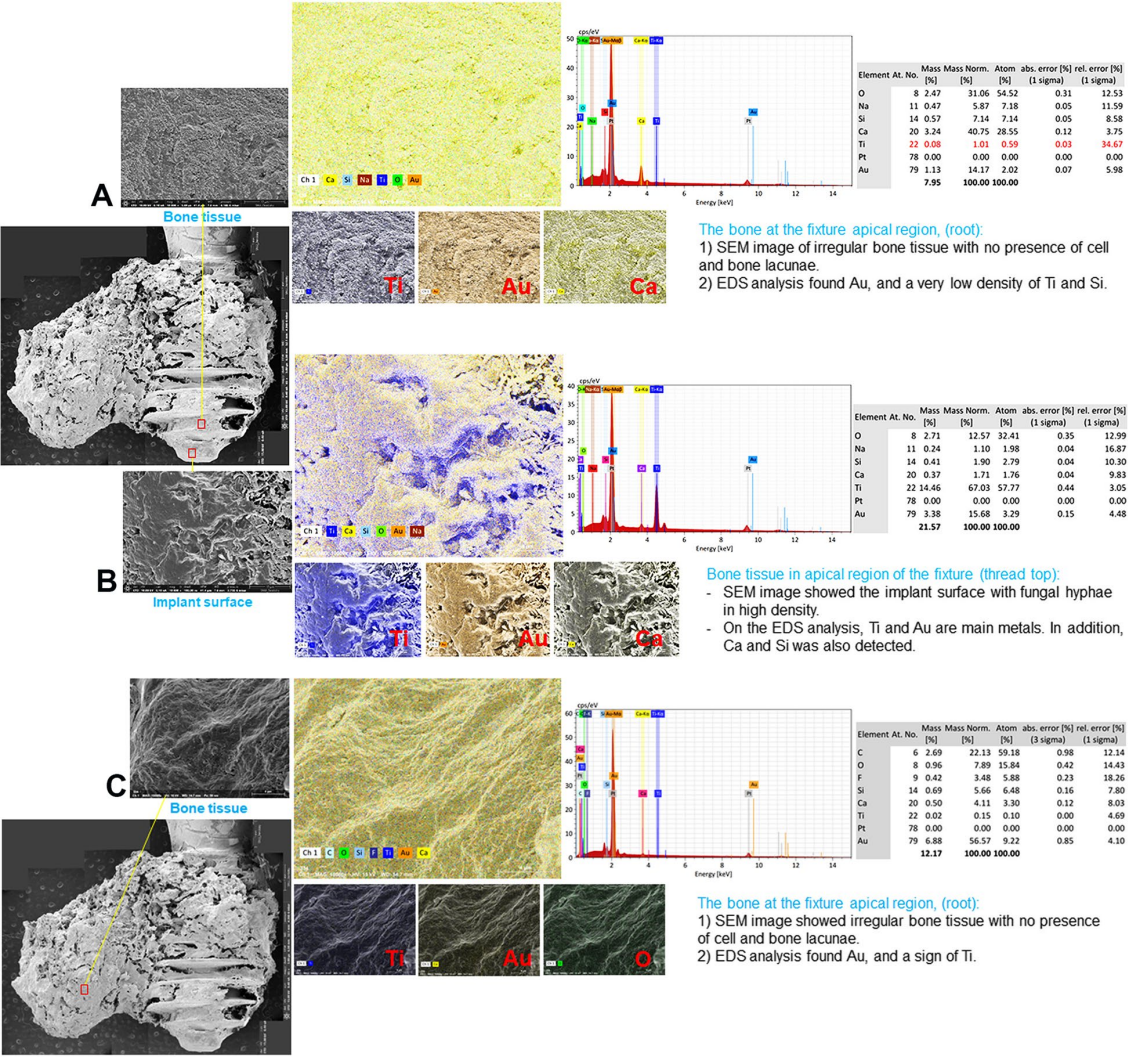
EDS results of patient D implant. The bone at the apical region of the fixture showed high levels of Ca (40.75%), O (31.06%), and Au (14.17%). Other detected elements were Si and Na, and a low signal of Ti was observed (1.01%) (**A**). The apical region of the fixture showed a significantly high percentage of Au (15.68%), Ti (67.03%), and O; Si, Ca, and Na were shown at low percentages (**B**). EDS analysis of the bone attached to the apical region of the bone exhibited a high percentage of Au (56.57%) and a low percentage of Ti (0.15%) (**C**)

The removed mass from patient D was confirmed to be chronic peri-implantitis, fungal sinusitis, and sequestrum. The high magnification micrograph was performed at five points: (1) at the bone tissue in the apical region of the implant, (2) at the exposed implant surface in the apical region of the implant, (3) at the necrotic bone tissue attached to the implant, (4) at another necrotic bone tissue attached to the implant, and (5) at implant surface near the upper region of the implant and were examined under 65 × , 500 × , 1000 × , 2500 × , 5000 × , 10,000 × , and 20,000 × magnifications. Points 1, 2, and 3 showed typical features of BRONJ with concave areas that show bone resorption pits seen on the bone surface of necrotic bone. The attached necrotic bone tissue on the implant surfaces of points 2 and 3 showed a pattern of sclerosing bone with rare signs of bone lacunae with several microcracks (Fig. 9). The EDS results of the bone at the apical region of the fixture showed high levels of Ca (40.75%), O (31.06%), and Au (14.17%). Other detected elements were Si and sodium (Na), and a low signal of Ti was observed (1.01%) (Fig. 10A). The EDS analysis at the apical region of the fixture revealed a significantly high percentage of Au (15.68%), Ti (67.03%), and O; Si, Ca, and Na were detected at low percentages in this region (Fig. 10B). In the EDS analysis of the bone attached to the apical region, exhibited a high percentage of Au (56.57%) and a low percentage of Ti (0.15%) (Fig. 10C).

## Discussion

Implant failure is caused by a variety of causes, which is a subject of interest to many clinicians and researchers. In addition to problems with the implant itself, placement, and loading, dropouts are occurring in relation to systemic diseases. Among them, BP, which is used to prevent excessive bone resorption in osteoporosis and cancer metastasis, is known as a drug that induces osteonecrosis, which may cause implant failure after tooth extraction in the jaw [12, 13]. According to the AAOMS, BRONJ is defined as follows: (1) present or a history of treatment with antiresorptive or antiangiogenic agents, (2) exposed bone or bone that can be probed through an intraoral or extraoral fistula in the maxillofacial region that has persisted for more than 8 weeks, and (3) no history of radiation therapy to the jaws or obvious metastatic disease to the jaws [11, 14–16].

BPs might be administered either orally or intravenously, and oral BPs, such as alendronate, are most frequently prescribed for osteoporosis and osteopenia [17]. Intravenous (IV) BPs, such as risedronate, pamidronate, and zoledronate, are not only effective for osteoporosis, but also for the treatment of hypercalcemia, multiple myeloma, metastatic cancer, and as an alternative in patients who cannot tolerate the gastrointestinal effects of oral BPs [6]. Four patients in our study, have received IV risedronate due to its frequent prescribed tendencies in Korean internal medicinist compared with those of other countries [18].

BRONJ, peri-implantitis, osteomyelitis, and osteoradionecrosis of the jaw are different entities and the etiology and the pathogenesis are of different origin. The pathogenesis of BRONJ starts with the fact that when the integrity of the oral mucosa of a patient taking BP is broken due to dental treatment, the microbiofilm formed on it penetrates [19]. According to Greg Wanger et al. [20], there is a bacterial nanowire that shows conductivity by special microorganisms, especially metal-producing bacteria, and it plays a more important role in penetration. The hypotheses of BP-induced bone destruction are, first, on the direct role of bone, and second, on the indirect effect on the permeability of the gingival epithelium [21]. Peri-implantitis, associated with severe biological complication, is defined as an inflammatory disease affecting tissues surrounding the implant and resulting in bone loss and eventually implant failure [22]. Osteomyelitis of the jaw may be induced either by hematogenous origin or by dissemination of local infection due to odontogenic infection or trauma [5]. Osteoradionecrosis of the jaw is defined as a complication of radiation that causes a disruption of vascular supply or avascular necrosis with bone exposure in jaw bones that fails to heal over a period of 3–6 months in the absence of local tumor recurrence [23].

One of the main distinct ultrastructural findings of the affected BRONJ specimen is the presence of a high number of microcracks [11]. Almost in all our ultrastructural findings through SEM analysis, a high number of microcracks were found in the attached BRONJ bone to the implant surface. Microcracks are defined as sharp edges larger than canaliculi but smaller in size compared to vascular canals [14]. In an animal study by Kim et al. [24], microcracks were found in SEM analysis of the BRONJ model in rats. The presence of microcracks can be explained by the fact that the jaw bone receives frequent loads with a high degree of mechanical stress by functional forces such as mastication that may lead to cracks. However, in healthy bone, these cracks are continuously repaired by the detection of osteocyte cells that transmit signals for repair [24]. In De Ponte et al.’s SEM study, the healthy bone showed the presence of bone lamellae parallel to each other and partially overlapping like roof tiles, alternating to bone lamellae with the same architecture, but with opposite orientation. Meanwhile, the BP-treated bone biopsy showed visible extensive and frequent areas consisting of a honeycomb structure, or areas with half-cells of different sizes and irregular boundaries, occasionally, partially overlapping each other [21]. Moreover, in Lee’s study, in the SEM observation using decalcified bone microsections, the normal bone showed interdigitating attachment of dendritic bone matrixes which were tightly arranged with each other. The dendritic bone matrixes were sequela of cytoplasmic processes of osteocytes, which contained organic bone matrixes and remained after the demineralization of the bone. The interdigitating dendritic bone matrixes produced many Haversian canaliculi, whereas the BP-involved bone showed granular bone matrixes which were more compact than the normal bone. The Haversian canaliculi formed between the granular bone matrixes were reduced in number and sometimes obliterated abortively [25]. Our main findings suggest that microcracks are one of the most distinct features of necrotic bone tissues found near the surface of the failed implants removed from the jaw of patients with BRONJ. The main ultrastructural findings of peri-implantitis are reported to be osteoclastic resorption lacunae, with altered osteocyte spaces that could be associated to the inflammatory process and the consequences of the increased loads on the remnant bone tissue [26]. For osteomyelitis of the jaw, bacterial biofilms with mixed species and the presence of resorption pits filled with bacterial biofilms are the distinct features found in the ultrastructural findings [27]. In the osteoradionecrosis of the jaw, microorganisms including rods, spirochetes, and cocci, with rods being the predominant cell were the distinct features in the SEM and TEM analysis [28]. Our study supports the previous findings that microcracks could be the first step in the pathogenesis of BRONJ, where a high number of microcracks in the bone samples from BRONJ were detected while samples from osteomyelitis and osteoradionecrosis did not present any microcracks [14]; therefore, the null hypothesis of this study would be rejected.

Our null hypothesis is rooted in the concept that irrespective of the underlying disease process, changes in the implant surface due to host response, biofilm formation, or altered local conditions could potentially exhibit certain similarities in terms of ultrastructural features. While the primary focus of our study is to explore the specific characteristics of implant surfaces in the context of BRONJ, we also recognize the potential significance of cross-comparisons with other conditions. However, we emphasize that our null hypothesis is based on the notion that certain ultrastructural changes might manifest regardless of the specific disease entity. It is important to note that our study aims to contribute insights into the ultrastructural aspects of failed implant surfaces in the context of MRONJ, and we acknowledge the complexity and variations between different disease entities. Future research could certainly explore further comparisons between these conditions to validate or refute our hypothesis.

According to Paulo et al., following tooth extraction in case of the chronic treatment with bisphosphonates, the inflammatory process leads to a decrease in pH, which favors the release of bisphosphonates from the bone reservoir to the surgical wound. This further inhibits the proliferation of fibroblasts, epidermal cells, and endothelial cells resulting in delayed closure of the mucosal barrier and prolonging the deleterious effects of exposure of the underlying bone to microorganisms [19].

Hoefert et al. [14, 29] evaluated the possible role of microcracks in the pathogenesis of BRONJ and discussed its causal model. In his study, SEM analysis found that 54% of BRONJ showed microcracks. In 82% of cases, inflammatory and connective tissue reactions were seen within microcracks. Only 29% of patients taking the medication without symptoms and 17% of osteoporotic patients showed microcracks, but not in osteomyelitis and osteoradionecrosis [14]. The reason microcracks occur in BRONJ is related to a decrease in bone remodeling induced by suppression of osteoclast function due to BP. If bacteria penetrate the generated crack, it becomes symptomatic ONJ [29]. Microcracks can be considered an “important first step” in the pathogenesis of ONJ [14, 29]. Kwon et al. [30] found that BRONJ occurring a short time after dental implant surgery would be regarded as a surgery-related complication. Kim et al. [24] compared the bony reversal lines seen in BRONJ and osteomyelitis. In this study, immature bony matrices outlined by thick reversal lines in BRONJ are evidence of rapid bone destruction osteonecrosis. These unrepaired microcracks were significantly associated with the development of BRONJ [24]. In our study, as in the above findings, microcracks were observed in the necrotic bone of patients taking BP, and bone resorption lacunae were also observed.

SEM and EDS is an effective tool for analyzing BP concentration in the jaw bone and provides important insight into BP pharmacokinetics and BRONJ. With SEM–EDS microanalysis, assessment and quantification of the presence of different bone types based on elemental analysis of Ca, phosphorous (P), and N were carried out. Four representative mineralization areas were found, considering the relative atomic Ca, P from the inorganic bone components, and N content from the organic bone component [12, 13, 31]. Therefore, for the analysis of necrotic bone and failed implants in BRONJ patients, a more effective research method was carried out in this study compared to the previously used method. The ultrastructural findings of BRONJ and implant surfaces were analyzed through SEM–EDS and TEM analysis. BPs, especially N-BP, mainly bind to hydroxyapatite bone minerals at the site of resorption and are captured in the osteoclast during bone destruction [8]. Therefore, N-BPs inhibit the prenylation of small guanosine triphosphate (GTP)-binding proteins in osteoclasts. This series of processes eventually lead to the loss of osteoclast function due to the destruction of the cytoskeleton. The main target cell of N-BPs is bone-resorbing osteoclasts; thus, numerous bone resorption lacunae on the surface of necrotic trabecular bone can be confirmed in our TEM findings, and this indicates that the bone resorption lacunae that occurred while the bone was alive are still present [8, 32].

In Aoki et al.’s study [11], the numbers of resorption lacunae and the length of the erosion on the bone surface of vital bones adjacent to the necrotic bones were increased, and these values in the necrotic bones were correlated with those of the vital bones in BRONJ. According to Kniha et al. [26, 33], the poor state of the osteoclast organelle shown in TEM findings indicates that it is less active or underdeveloped. This is different from the osteocytes seen in the hardened bone or the osteoblasts seen at the edge of the mineralized bone. In Christian Gross’s [34] study, osteoclast inactivation and high cell-to-cell fusion rate were found in the osteoclast profile of MRONJ, and the presence of giant, hypernucleated osteoclasts cannot be attributed to increased dendritic cell-specific transmembrane protein (DC-STAMP) triggered cell-to-cell fusion alone. Our previous study [35] also found that dendritic cells and titanium particles were seen in the necrotic bone removed with peri-implantitis. Peri-implantitis is an inflammatory response, and macrophage-like antigen-presenting cells (APCs) migrate around the inflamed impeller. The dendritic cell (DC) is a member of the APC and is known to initiate and regulate immune response to foreign antigens [35, 36]. However, in the necrotic bone of our study, dendritic cells are not visible.

The absence of dendritic cell in our specimens can be explained by the finding of Elsayed et al. in vitro study [36] stating that BP, especially *N* = BP inhibit the differentiation and function of dendritic cell rendering the microenvironment more conducive to bacterial colonization and subsequent osteonecrosis. Taking into account, most patients in our study consumed BP for more than 1 year suggesting the high accumulation of BP may have severely suppressed the differentiation of dendritic cells.

Through SEM and EDS analysis, titanium particles were found all over the implant surface in various studies. Shibli’s [37] SEM analysis showed four different degrees of organic residues, appearing mainly as dark stains. The surface topography showed grooves and ridges along the machined surface similar to that of the control group. Overall, foreign elements, such as Ca, O, Na, C, Si, and aluminum (Al), were detected in failed implants. The implants from the control group presented no macroscopic contamination and clear signs of Ti. Nguyen et al. [12] studied the surface of the removed implant which was examined in a patient with maxillary sinusitis caused by various causes. Among them, SEM findings at the apex of the removed implants in BRONJ patients showed no cells or lacuna on the irregular bone surface.

Noumbissi et al. [38] showed that metal ions are released from titanium alloy dental implants due to corrosion. The presence of the long-term corrosion not only leads to the release of ions into the peri-implant tissue but also a disintegration of the implant that contributes to material fatigue and even fracture of the abutments, implant body, or both. From our recent study [15, 16], Ti, C, and O from EDS analysis are not harmful elements due to the chemical composition of the implant. However, inorganic impurities such as Al, zinc (Zn), Si, and magnesium (Mg), with other elements such as nitrogen (N), F, P, Cl, and Na contribute to the corrosion process.

The various metals used in the alloy used in the implant—copper (Cu), Al, silver (Ag), vanadium (V), and manganese (Mn)—are associated with high cytotoxicity and reduced cell viability. According to Park et al., the following elements are in decreasing cytotoxity: Cu > Al > Ag > V > Mn > chromium (Cr) > zirconium (Zr) > niobium (Nb) > molybdenum (Mo) > commercial pure Ti (CP-Ti) [39]. Currently, biomedical Ti is available in four commercially pure grades (ASTM I-IV) and several alloys, including Ti-6Aluminum (Al)-4Vanadium (V) (Ti6Al4V; ASTM Grade V). For the four grades of Coptic, ISO 5832–2 and F67-13 specify alongside Ti, the maximum elemental mass fractions of nitrogen (N) (max.: 0.012–0.05 mass %), carbon (C) (max.: 0.03–0.08 mass %), hydrogen (H) (max.: 0.0125 mass %), oxygen (O) (max.: 0.1–0.4 mass %), and iron (Fe) (max.: 0.1–0.5 mass %) contents. The Fe and O fractions increase from Grade I to Grade IV Ti and correlate with the enhancement of the hardness, yield, and tensile strengths but a decrease in corrosion resistance. The elemental composition of Grade IV Ti, the most common commercially pure Grade of Ti used in dental implants, is standardized as follows: N: max. 0.05 mass%; C: max. 0.08 mass%; H: max. 0.0125 mass%; Fe: max. 0.5 mass%; O: max. 0.4 mass%; and Ti: balance. No other metal element fractions are specified or limited for CpTi in the respective standards [40]. Au element is mainly found in the abutment or prosthesis of the implant. The connection between the implant fixture and abutment may result in the release of metal ions. Ti behaves differently when connected to different materials; it acts as an anode when connected to a noble metal such as Au, whereas it is considered the cathode when connected to a base metal [41]. Therefore, in this study, a high percentage of Au is doubtlessly due to galvanic current activity from gold abutment or corroded gold prosthesis in the mastication during mastication, not from coating material, and can be regarded as contributing factor for periimplantitis, especially in patients with compromised bone tissue such as patients with BRONJ. Al nanoparticles act on the immune system and affect not only immune organs but also immune cells [42, 43].

Dental implant-related systemic toxicity of Al nanoparticles is not known. However, it seems to induce an inflammatory response of the Schneiderian membrane by locally inducing immune cell dysfunction and abnormal immune-related cytokine behavior [44, 45]. EDS analysis in our study revealed that in addition to the main titanium element, gold, carbon, oxygen, calcium, phosphorus, and silicon elements were found. Furthermore, it was also revealed that sulfur was found, which was considered to be one of the complicated causes of implant failure in BRONJ patients. Arteaga et al. [46] tested Ti in an environment similar to diabetes, and there was also an increase in Al. Guler et al. [47] compared the failed implant surfaces and looked at the differences between implant types. In his study, C, N, Ca, P, Cl, S, Na, and Si were also released from a titanium oxide layer on the implant surface. The sulfur (S) component present on the implant surface may be related to the end products of the microorganisms. In our case, S was detected in SEM/EDS analysis; however, it may be that S in our study is not necessarily due to BPs, but the complex microorganisms.

## Conclusions

Hardened bone tissues with microcracked bony resorbed lacunae were observed in the SEM findings, which were considered as the main characteristic of the osteonecrosis of the jaw. Immune cells, such as DCs, in the failed implant surface of the BRONJ-related peri-implantitis tissues were not identified in the TEM investigations. EDS analysis showed that in addition to the main Ti element, Au, C, O, Ca, P, and Si elements were found. In addition, the finding of sulfur, inflammatory, and bacterial byproducts are considered as the contributing factors to the implant failure in patients with BRONJ.

## Data Availability

This study was conducted as a retrospective research method, and raw data can be provided at the request of the journal.

## Abbreviations

BP: Bisphosphonates
BRONJ: Bisphosphonate-related osteonecrosis of the jaw
EDS: Energy dispersive X-ray spectroscopy
GA: Glutaraldehyde
MRONJ: Medication-related osteonecrosis of the jaw
N-BP: Nitrogen-containing BP
SEM: Scanning electron microscope
TEM: Transmission electron microscope
